# Supplementary material for: The association between inadequate sleep and accelerated brain ageing
Source: Neurobiol Aging. 2022 Jun;114:1–14. doi: 10.1016/j.neurobiolaging.2022.02.005 (PMC9084918; doi:10.1016/j.neurobiolaging.2022.02.005)
Supplement: Supplementary file 1 [file mmc1.docx]

**SUPORTING INFORMATION**

**Supplementary Table 1. Complete list of the 110 T1 IDPs that correspond to the volumes of the cortical and subcortical ROIs (GM ROIs) derived from the Harvard-Oxford structural atlases.**

| ID | GM ROI | ID | GM ROI |
| --- | --- | --- | --- |
| 1-2 | Bilateral Frontal Pole | 56-57 | Bilateral Cingulate Gyrus (anterior) |
| 3-4 | Bilateral Insula Cortex | 58-59 | Bilateral Cingulate Gyrus (posterior) |
| 5-6 | Bilateral Superior Frontal Gyrus | 60-61 | Bilateral Precuneus |
| 7-8 | Bilateral Middle Frontal Gyrus | 62-63 | Bilateral Cuneal Cortex |
| 9-10 | Bilateral Inferior Frontal Gyrus (pars triangularis) | 64-65 | Bilateral Frontal Orbital Cortex |
| 11-12 | Bilateral Inferior Frontal Gyrus (pars opercularis) | 66-67 | Bilateral Parahippocampal Gyrus (anterior) |
| 13-14 | Bilateral Precentral Gyrus | 68-69 | Bilateral Parahippocampal Gyrus (posterior) |
| 15-16 | Bilateral Temporal Pole | 70-71 | Bilateral Lingual Gyrus |
| 17-18 | Bilateral Superior Temporal Gyrus (anterior) | 72-73 | Bilateral Temporal Fusiform (anterior) |
| 19-20 | Bilateral Superior Temporal Gyrus (posterior) | 74-75 | Bilateral Temporal Fusiform (posterior) |
| 20-21 | Bilateral Middle Temporal Gyrus (anterior) | 76-77 | Bilateral Temporo-occipital Fusiform |
| 22-23 | Bilateral Middle Temporal Gyrus (posterior) | 78-79 | Bilateral Occipital Fusiform Gyrus |
| 24-25 | Bilateral Middle Temporal Gyrus (temporo-occipital) | 80-81 | Bilateral Frontal Operculum |
| 26-27 | Bilateral Inferior Temporal Gyrus (anterior) | 82-83 | Bilateral Central Operculum |
| 28-29 | Bilateral Inferior Temporal Gyrus (posterior) | 84-85 | Bilateral Parietal Operculum |
| 30-31 | Bilateral Inferior Temporal Gyrus (temporo-occipital) | 86-87 | Bilateral Planum Polare |
| 32-33 | Bilateral Postcentral Gyrus | 88-89 | Bilateral Heschl’s Gyrus |
| 34-35 | Bilateral Superior Parietal Lobule | 90-91 | Bilateral Planum Temporale |
| 36-37 | Bilateral Supramarginal Gyrus (anterior) | 92-93 | Bilateral Supracalcarine Cortex |
| 38-39 | Bilateral Supramarginal Gyrus (posterior) | 94-95 | Bilateral Occipital Pole |
| 40-41 | Bilateral Angular Gyrus | 96-97 | Bilateral Thalamus |
| 42-43 | Bilateral Lateral Occipital Superior | 98-99 | Bilateral Caudate |
| 44-45 | Bilateral Lateral Occipital Inferior | 100-101 | Bilateral Putamen |
| 46-47 | Bilateral Intracalcarine Cortex | 102-103 | Bilateral Pallidum |
| 48-49 | Bilateral Frontal Medial Cortex | 104-105 | Bilateral Hippocampus |
| 50-51 | Bilateral Juxtapositional Lobule | 106-107 | Bilateral Amygdala |
| 52-53 | Bilateral Subcallosal Cortex | 108-109 | Bilateral Ventral Striatum |
| 54-55 | Bilateral Paracingulate Gyrus | 110 | Brainstem |
